# Preserved right ventricular function but increased right atrial contractile demand in altitude-induced pulmonary hypertension

**DOI:** 10.1007/s10554-020-01803-x

**Published:** 2020-03-09

**Authors:** Mahdi Sareban, Tabea Perz, Franziska Macholz, Bernhard Reich, Peter Schmidt, Sebastian Fried, Heimo Mairbäurl, Marc M. Berger, Josef Niebauer

**Affiliations:** 1 University Institute of Sports Medicine, Prevention and Rehabilitation and Research Institute of Molecular Sports Medicine and Rehabilitation, Paracelsus Medical University, Lindhofstraße 20, 5020 Salzburg, Austria; 2 Department of Anesthesiology, Perioperative and General Critical Care Medicine, Salzburg General Hospital, Paracelsus Medical University, Müllner Hauptstraße 48, 5020 Salzburg, Austria; 3 Medical Clinic VII, Sports Medicine; Translational Lung Research Center Heidelberg (TLRC-H); Member of the German Center for Lung Research (DZL), University of Heidelberg, Im Neuenheimer Feld 410, 69120 Heidelberg, Germany; 4 Department of Anesthesiology, University Hospital Heidelberg, Im Neuenheimer Feld 110, 69120 Heidelberg, Germany

**Keywords:** Altitude, Pulmonary hypertension, Echocardiography, Atrial function

## Abstract

**Purpose:**

Ascent to high altitude increases right ventricular (RV) afterload and decreases myocardial energy supply. This study evaluates physiologic variables and comprehensive echocardiographic indices of RV and right atrial (RA) function following rapid ascent to high altitude.

**Methods:**

Fifty healthy volunteers actively ascended from 1130 to 4559 m in < 22 h. All participants underwent 2D echocardiography during baseline examination at low altitude (424 m) and at three study time-points (7, 20 and 44 h) after arrival at high altitude. In addition to systolic pulmonary artery pressure (sPAP), comprehensive 2D planimetric-, tissue Doppler- and speckle-tracking-derived strain indices of RA and RV function were obtained.

**Results:**

sPAP increased from baseline (24 ± 4 mmHg) to the first altitude examination (39 ± 8 mmHg, *p* < 0.001) and remained elevated during the following 44 h. Global RV function did not change. RA reservoir strain showed a trend towards increase from baseline (50.2 ± 12.1%) to the first altitude examination (53.8 ± 11.0%, *p* = 0.07) secondary to a significant increase of RA contraction strain (19.2 ± 6.4 vs. 25.4 ± 9.6%, *p* < 0.001). Volumetric RA data largely paralleled RA strain results and RA active emptying volume was increased throughout the 44 h stay at high altitude.

**Conclusion:**

Active and rapid ascent of healthy individuals to 4559 m is associated with an increased contractile performance of the RA that compensates for the increased workload of the RV.

**Electronic supplementary material:**

The online version of this article (10.1007/s10554-020-01803-x) contains supplementary material, which is available to authorized users.

## Introduction

High-altitude exposure causes several physiological responses of the pulmonary and cardiovascular system, such as increase in pulmonary artery pressure (PAP) leading to an increase in right ventricular (RV) afterload [1, 2]. Whereas individuals with well-documented altitude tolerance show a moderate increase in PAP (38 ± 3 mmHg) [3], a more pronounced increase seen in mountaineers susceptible for high-altitude pulmonary edema (48 ± 10 mmHg) [3] leads to an even higher RV workload. In case of relevant RV dysfunction, including impairment of systolic and diastolic function, right-heart failure may occur [4]. The role of the right atrium (RA) in compensating altitude-induced pulmonary hypertension has received little scientific attention to date. This may be explained by technical limitations, precluding a comprehensive echocardiographic assessment of RA mechanics in the past. The RA, however, plays an integral role in cardiac performance by improving RV filling with three repetitive consecutive functional phases [8]: during the *reservoir phase* the RA collects central venous return. During the subsequent *conduit phase* the RA passively channels blood into the RV as the tricuspid value opens during RV diastole. During the final period of RV relaxation, the *contraction phase (atrial systole)* of the RA actively pumps blood into the RV. 2D-Transthoracic echocardiography (TTE) enables volumetric determination of phasic RA function and can be even performed in field conditions at high altitude. Recently, the advent of 2D speckle tracking echocardiography (STE)-derived strain analysis has added less angle-, load and cardiac translation-influenced information about intrinsic myocardial function and reliability of this technique has already been reported for RA analysis [9].

Shedding light on RA function at high altitude has the potential to improve the understanding of mechanisms necessary for compensating for an altitude-induced increase in RV workload. In addition, echocardiographic assessment of the RA prior to ascending to high altitude may improve risk stratification for acute high-altitude illnesses. Consequently, and in view of rising interest in both recreational as well as occupational high-altitude activities, we aimed to investigate both the RV and RA structural and functional response following rapid and active ascent of healthy individuals to high altitude. Therefore, classic volumetric-, Doppler-derived, and novel STE-derived RV and RA strain variables were assessed at low altitude (424 m) and at three time points after active and rapid ascent to 4559 m.

## Methods

### Study approval, registration and population

The study was performed in accordance with the Declaration of Helsinki and its current amendments, and was approved by the Ethical Committee Salzburg, Austria, by the Ethical Committee of the University of Torino, Italy, and by the Competent Authority (BASG), Vienna, Austria. This study was part of a trial showing that inhaled budesonide did not prevent acute mountain sickness (AMS) as detailed elsewhere [10]. Because budesonide inhalation did not have any significant effect on main variables of RV function [11], the data from all subjects were pooled for the present analyses. Data on intra- and interobserver variability of strain measurements of the RA and LA as well as measurements of LV and LA function have already been published.

Following ethical approval, 50 predominantly physically fit male, healthy, non-smoking native lowlanders were recruited via social media and public postings. None of these subjects had spent time at altitudes > 2000 m within the last 4 weeks before the study and none took any regular medication. Subjects with cardiovascular diseases were excluded. All subjects provided written informed consent before study inclusion.

### Study design

We performed baseline measurements including exercise testing and echocardiographic assessment at an altitude of 424 m (Salzburg, Austria). Between 2 to 4 weeks after baseline examination, subjects travelled to Alagna (1130 m), Valsesia, Italy, and ascended in groups of 10 accompanied by licensed mountain guides to 4559 m within 20 h. The ascent consisted of transport by cable car to 3275 m, and a 90-min climb to 3611 m (Capanna Giovanni Gnifetti), where they spent one night. On the next morning, subjects performed a 4 to 5 h climb to 4559 m (Capanna Regina Margherita, Monte Rosa).

### Echocardiography

All participants underwent TTEs during baseline examination and 7, 20 and 44 h after arrival at 4559 m using a commercially available ultrasound system (Philips CX50, Phillips Medical Systems, Andover, MA, USA) with a 1.0–5.0 MHz sector array transducer (Philips S5-1, Phillips Medical Systems, Andover, MA, USA). All TTEs were conducted by the same experienced cardiac sonographer with the subject lying in the left lateral decubitus position after participants rested for five minutes in supine position. An electrocardiogram connected to the ultrasound system recorded heart rate (HR) during the examination. Single beat calculations were used to calculate volumetric and speckle-tracking derived data. All images were saved in a raw Digital Imaging and Communications in Medicine (DICOM) format on a mass storage device and analyzed offline using commercially available software (Philips Xcelera, Phillips Medical Systems, Andover, MA, USA).

#### *Systolic pulmonary artery pressure and stroke volume analysis*

As shown previously Doppler echocardiography represents an accurate and reproducible method for estimating systolic pulmonary artery pressure (sPAP) at high altitude and correlates well with invasive measurements [12]. For the determination of sPAP, peak-flow velocities of tricuspid valve regurgitation jets using continuous wave Doppler were measured as detailed elsewhere [13]. For calculation of sPAP, 5 mmHg were added as an estimate for right atrial pressure [14]. Biplane LV volumes were measured at end-diastole (LVEDV) and end-systole (LVESV) allowing for calculation of cardiac stroke volume (SV) using the summation of disks method [15].

#### *Structural and functional RV analysis*

Parameters of RV structure and function were assessed according to current international recommendations [13]. Proximal right ventricular outflow tract (RVOT) diameter was measured in the basal parasternal short-axis view. The basal and mid cavity RV diameters, as well as the RV longitudinal dimension were obtained in the apical four chamber view.

The RV fractional area change (FAC), a global estimate of systolic RV function, was assessed by tracing the endocardial RV border in a RV-focused apical 4-chamber view at end-diastole and end-systole. Trabeculation, tricuspid leaflets, and chords were included in the chamber and FAC calculated by using the formula: end-diastolic area − end-systolic area)/end-diastolic area × 100 [13].

Tricuspid annular plane systolic excursion (TAPSE), a measure of longitudinal RV systolic function, was assessed by placing a M-mode beam through the lateral tricuspid annulus and the highest amount of RV longitudinal motion of the annulus at peak systole was obtained from a standard apical 4-chamber window [13]. Peak tricuspid lateral annular systolic velocity (s′), a measure of global RV systolic function, was assessed by placing the pulsed wave Tissue-Doppler sample volume in the lateral tricuspid annulus of the RV free wall using a standard apical 4-chamber window. The velocity s’ was read as the highest systolic velocity without over-gaining the Doppler envelope [13]. The RV myocardial performance index (MPI), a global estimate of both systolic and diastolic RV function and also known as Tei index, was assessed using the same pulsed wave Tissue-Doppler image as s’ and calculated as the sum of RV isovolumic contraction and relaxation times divided by ejection time [13].

#### *Structural and functional RA analysis*

**Fig. 1:**
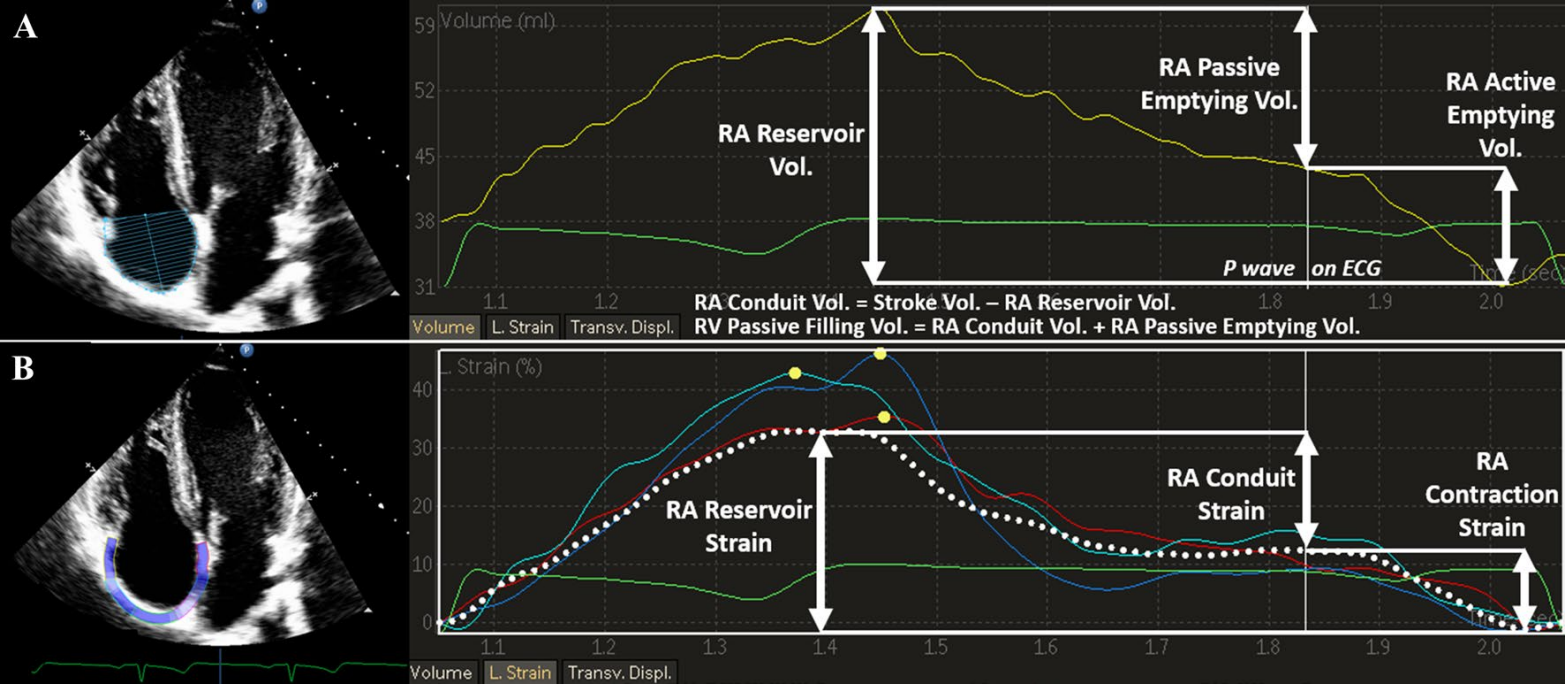
**A** Right atrial functional phases during a cardiac cycle assessed by volumetric-derived measurements. **B** Right atrial functional phases during a cardiac cycle assessed by speckle tracking-derived strain using QRS timed analysis. Dotted line depicts average curve of the six segments

RA volumes were measured at three time points using the single-plane (apical 4-chamber) method of disks as described in Fig. 1: (i) the maximal volume (RAV_max_) just before the opening of the tricuspid valve, (ii) pre-atrial contraction volume (RAV_preA_), obtained at the onset of the P-wave on surface electrocardiogram, and (iii) the minimal volume (RAV_min_) at the closure of the tricuspid valve. RA reservoir volume (i.e. RA stroke volume (RASV)) was calculated as the difference between RAV_max_ and RAV_min_, RA passive emptying (i.e. early RV diastolic filling) volume as the difference between RAV_max_ and RAV_preA_, and RA active emptying (i.e. late RV diastolic filling) volume as the difference between RAV_preA_ and RAV_min_, respectively. We calculated conduit volume, representing the flow from the caval veins to the RV, as the difference between cardiac SV and RA reservoir volume. RAV_max_ indexed (RAVI) to body surface area (BSA) was measured and BSA was derived from the formula of Mostseller: BSA = 0.01666667 body height^−0.5^ body weight^−0.5^.

#### *Speckle tracking-derived RV and RA analysis*

Images for longitudinal STE-derived strain analysis of RV and RA derived from RV and RA focused apical 4-chamber views. The sonographer optimized image quality by positioning the focus at the region of interest and adjusting sector depth and width to include as little as possible outside the region of interest in order to maintain a frame rate between 50 and 80/min. The same investigator performed offline analyses using a commercially available acoustic tracking software package (QLAB 10.3 (cardiac motion quantification (CMQ) for RV strain, QLAB version 9.0 for all other strain measurements; Phillips Medical Systems, Andover, MA, USA)). Thereby, the region of interest was set at the myocardium using a point-and-click technique and the software automatically tracked the endocardial contour. Segments in which inadequate tracing was observed were excluded from further analysis. Before processing, a cine loop preview was used to confirm that speckles remained within the myocardium throughout the cardiac cycle and additional manual adjustment was performed when myocardial tracking was unsatisfactory.

Peak RV free wall longitudinal strain was obtained from three segments of the RV free wall and the segments were averaged.

For RA strain analysis, the frame at the onset of the R-wave was used as the reference frame. The software divided the RA wall into six equidistant segments and the segments were averaged for further analysis. Peak, pre-atrial contraction and minimal strain values were derived from the maximal inflection point, the point correlating with the onset of the P-wave on surface ECG and the minimal inflection point on the RA strain curve, respectively. Consequently, 2D-STE-derived atrial reservoir-, conduit- and contractile-strains were calculated as illustrated in Fig. 1. We already demonstrated high intra- as well as inter-observer reliability of these variables in our laboratory [9].

### Further measurements

After 5 min of rest in supine position peripheral oxygen saturation (SpO_2_) was measured by pulse oximetry (Covidien Nellcor, Mansfield, USA), and mean arterial blood pressure (MAP) was measured non-invasively by analysing brachial artery waveforms (Pulsecor, Auckland, New Zealand).

### Statistical analysis

Continuous variables are presented as means ± SD. Normal distribution of the data was tested using the Kolmogorov–Smirnov test. Effects of time were analyzed by 1-way-repeated measures ANOVA and effects of altitude and treatments by 2-way-repeated-measures ANOVA. Adjustments for multiple comparisons were performed with the Bonferroni correction. The relationship between pairs of variables was expressed with the Pearson (*r*) Correlation Coefficient and effect sizes of correlation were defined as follows: trivial, 0.0; small, 0.1–0.3; moderate, 0.3–0.5; large, 0.5–0.7; very large 0.7–0.9; nearly perfect 0.9, and perfect 1.0 [16]. A *p*-value ≤ 0.05 (two-sided) was considered to be significant.

Regarding correlation analysis, only statistical significant results with at least moderate effect size are mentioned in the results. All statistical analyses were performed using SPSS 24 for Windows (SPSS, Inc., Chicago, IL).

## Results

Baseline characteristics of the participants are listed in Table 1. 62% of subjects developed AMS. Four subjects developed severe AMS and were excluded from the study because of necessary medical treatment. Thus, data of 46 participants were available for final analyses.

**Table 1:** Physical characteristics of the study population

Variables	n = 46
Male	34
Age (years)	36 ± 11
Body height (cm)	176 ± 9
Body weight (kg)	72 ± 10
BMI (kg/m^2^)	20.3 ± 2.3
BSA (m^2^)	1.9 ± 0.2
HR at rest (beats/min)	64 ± 11
Systolic BP at rest (mmHg)	123 ± 12
Diastolic BP at rest (mmHg)	72 ± 7
Maximal exercise capacity (W/kg)	4.2 ± 0.5

Values are presented as arithmetic mean ± SD or number of participants

*BMI* body mass index, *BSA* body surface area, *HR* heart rate, *BP* blood pressure, *W* Watt

### Physiological characteristics

Tricuspid regurgitation jets could be displayed and consequently sPAP obtained in 46 out of 50 studies at baseline and 189 out of 191 at altitude. SpO_2_ decreased at high altitude whereas sPAP, HR and MAP increased following ascent and remained elevated at high altitude (Table 2). SpO_2_ correlated inversely with sPAP (*r* = − 0.58; *p* < 0.001), with RA active emptying volume (*r *= − 0.36; *p* < 0.001), and with HR (*r *= − 0.42; *p* < 0.001).

**Table 2:** Physiologic variables at low- and high altitude

	Low Alt	High Alt-7 h	High Alt-20 h	High Alt-44 h
Heart Rate (beats/min)	64.2 ± 11.2	79.1 ± 14.2***	76.7 ± 14.5***	75.3 ± 14.6***
SpO_2_ (%)	97.5 ± 1.3	77.5 ± 7.0***	81.1 ± 6.2***	81.5 ± 7.3***
sPAP (mmHg)	24.4 ± 3.8	38.5 ± 8.2***	38.2 ± 8.9***	38.0 ± 8.1***
MAP	89 ± 6	92 ± 7*	91 ± 6*	92 ± 5**
LV stroke volume	64.5 ± 15.0	58.1 ± 16.4*	60.4 ± 18.1*	61.2 ± 18.4*

Values are presented as arithmetic mean ± SD

*SpO*_*2*_ peripheral oxygen saturation, *sPAP* systolic pulmonary artery pressure, *MAP* mean arterial pressure, *LV* left ventricle, *Low Alt* low altitude (424 m), *High Alt* high altitude (4559 m)

*p < 0.05, **p < 0.01 and ***p < 0.001 vs Low Alt

### Echocardiographic characteristics

#### *Right ventricular characteristics*

Cardiac SV as assessed by LV SV decreased after ascent to high altitude (Table 1). RVOT increased whereas other structural RV dimensions as well as diastolic- and systolic RV areas remained unchanged (Table 3). TAPSE and s’ increased at high altitude whereas longitudinal strain did not change. Furthermore, the global RV parameters MPI and FAC did not change (Table 3).

**Table 3:** Echocardiographic variables of right ventricular function at low- and high altitude

	Low Alt	High Alt-7 h	High Alt-20 h	High Alt-44 h
RV geometric parameters				
RVOT prox. dimension (mm)	34.3 ± 5.3	36.5 ± 5.7***	37.2 ± 5.4***	37.0 ± 5.4***
RV basal cavity dimension (mm)	37.8 ± 5.5	39.5 ± 5.8	39.4 ± 4.8	38.6 ± 4.7
RV mid cavity dimension (mm)	31.0 ± 5.5	32.3 ± 5.4	31.8 ± 5.8	31.3 ± 5.3
RV longitudinal dimension (mm)	85.9 ± 9.6	86.7 ± 10.2	87.9 ± 10.6	87.8 ± 9.3
RV end-diastolic area (cm^2^)	21.8 ± 5.0	23.1 ± 5.8	22.2 ± 5.4	22.0 ± 5.5
RV end-systolic area (cm^2^)	10.8 ± 3.2	11.8 ± 3.5	11.4 ± 3.6	11.4 ± 3.9
RV functional parameters				
TAPSE (mm)	23.3 ± 3.7	25.0 ± 3.1	25.7 ± 2.7**	25.1 ± 3.3
FAC (%)	50.8 ± 6.5	49.5 ± 6.5	48.9 ± 8.7	48.7 ± 8.4
RV s′ (cm/s)	12.9 ± 1.8	14.0 ± 2.0*	14.1 ± 2.1*	14.2 ± 1.8*
MPI	0.40 ± 0.10	0.40 ± 0.14	0.40 ± 0.14	0.39 ± 0.11
RV strain (%)	25.4 ± 4.2	26.1 ± 4.2	26.1 ± 4.0	26.4 ± 4.4
RA geometric parameters				
RAVI_max_ (mL/m^2^)	26.3 ± 7.4	31.5 ± 8.9**	30.7 ± 8.9*	28.3 ± 8.0
RA_max_ volume (mL)	50.4 ± 17.3	59.8 ± 20.8**	59.0 ± 20.3*	54.0 ± 18.3
RA_preA_ volume (mL)	33.8 ± 12.5	40.2 ± 15.9**	39.4 ± 14.3**	35.4 ± 14.2
RA_min_ volume (Ml)	22.3 ± 8.7	22.7 ± 10.4	21.0 ± 8.4	19.7 ± 9.8*
RA functional parameters				
RA stroke volume (mL)	27.6 ± 11.1	37.1 ± 12.7**	37.2 ± 13.7***	34.0 ± 10.6**
RA reservoir strain (%)	50.2 ± 12.1	53.8 ± 11.0	54.6 ± 12.8	54.6 ± 11.9
RA conduit strain (%)	31.0 ± 9.3	28.4 ± 9.5	30.1 ± 9.6	31.7 ± 10.3
RA contraction strain (%)	19.2 ± 6.4	25.3 ± 9.6***	24.7 ± 9.5**	22.9 ± 7.7

Values are presented as arithmetic mean ± SD

*RVOT *right-ventricular outflow tract (from parasternal short axis view), *RV* right ventricle, *TAPSE* tricuspid annular plane systolic excursion, *FAC* fractional area change, *s*′ pulsed-wave Doppler tissue imaging (DTI)-derived peak systolic myocardial velocity (averaged from basal septum and basal lateral wall), *MPI* myocardial performance index, *RAVI* right atrial volume indexed to body surface are, *RA* right atrium, *Low alt* low altitude (424 m), *High alt* high altitude (4559 m)

*p < 0.05, **p < 0.01 and ***p < 0.001 vs Low Alt

#### *Right atrial characteristics*

**Fig. 2:**
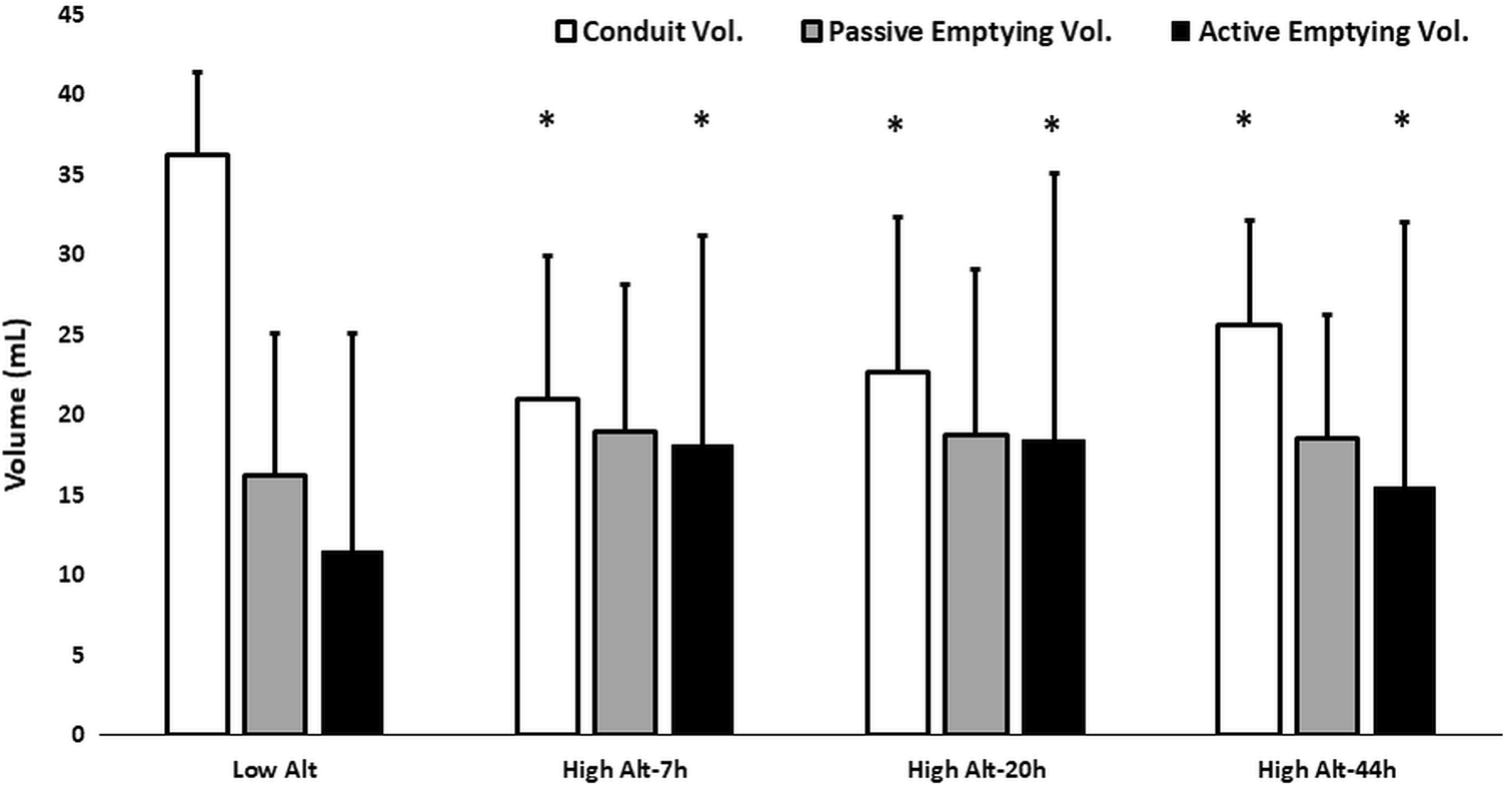
Right atrial phasic volumes from baseline examinations at low altitude (Low Alt), 7 h (High Alt-7 h), 20 h (High Alt-20 h) and 44 h (High Alt-44 h) after arrival at high altitude, respectively.*p < 0.001 vs Low Alt

Among the volumetric RA indices, RA_max_- and RA_preA_-volume were increased until 20 h after arrival at high altitude (Table 3). RA active emptying volumes increased at high altitude and remained elevated until the last assessment at 44 h after arrival at altitude (Fig. 2). RA conduit volume decreased at high altitude whereas passive emptying volumes did not change significantly (Fig. 2). RA reservoir strain showed a trend towards increase from baseline to the first altitude examination (53.8 ± 11.0%, *p* = 0.07) secondary to a significant increase of RA contraction strain which remained increased until 20 h after arrival at altitude (Table 3). RA conduit strain did not change upon altitude exposure (Table 3).

#### *Acute mountain sickness and echocardiographic variables of cardiac function*

There was no statistically significant group difference (all time points) or interaction effect (group*time) for echocardiographic variables between subjects with and without AMS (Supplemental Table 1). Echocardiographic variables did not differ at baseline between groups.

### Correlation analysis

HR correlated with moderate effect size with RA contraction strain (*r* = 0.5; *p* < 0.001) and s’ (*r* = 0.45; *p* < 0.001). sPAP correlated with moderate effect size with RA active emptying volume (*r* = 0.35; *p* < 0.001) whereas SpO_2_ correlated inversely with RA active emptying volume (*r* = − 0.36; *p* < 0.001).

## Discussion

The present study shows that after active and rapid ascent of healthy individuals to 4559 m global RV function is preserved but associated with an increased contractile workload of the RA. These findings may be of particular interest for people with pre-existing or increased risk for right-heart pathology.

Data regarding the effect of acute high-altitude exposure on RV function and structure are sparse and conflicting. While some studies report that an acute hypoxia-induced increase in RV afterload leads to an improved contractility [17], others found no change [18] or even an impaired contractility [19–21]. The discrepancies in the existing literature could be related to the considerable differences between methods and research designs. In the present study, we used a multitude of parameters for RV function assessment as suggested in recent recommendations [13] and we found that some longitudinal RV contractility parameters, i.e. TAPSE and s’, increased upon altitude exposure. This increase in contractility might be due to sympatho-activity as we observed a correlation between HR and s’. However, global RV contractility parameters, i.e. FAC, as well as longitudinal RV strain did not change. In addition to the highest number of study participants undergoing echocardiographic RV assessment at high altitude, our study extends the abovementioned studies, as we performed serial assessment of RV function at 4559 m indicating that the abovementioned functional changes are maintained for up to 44 h after arrival at high altitude. These results are in line with data on LV function of the same study cohort where we did not observe relevant LV systolic or diastolic dysfunction following ascent to 4559 m [22].

In contrast to the RV, the RA has a simpler geometric shape facilitating echocardiographic evaluation. In addition, it has an integral role in modulating RV filling and thus overall cardiac performance by its phasic function which can be assessed by volumetric and recently by speckle-tracking-derived quantification [8] with high reliability under different hemodynamic conditions, as recently demonstrated by our group [9]. To the best of our knowledge, this is the first study assessing RA geometric and functional changes at high altitude. Our results indicate that passive emptying volume is reduced secondary to a marked decrease of conduit volume. Although, active emptying volume is increased it cannot compensate for the passive filling reduction contributing to a reduced stroke volume. These volumetric changes are in line with our speckle-tracking-derived strain results, as RA-contraction strain was significantly increased up to 20 h after arrival at high altitude. Mechanistically, this might be due to the increased RA_preA_-volume/dimension or heterometric adaptation that we observed, leading to an increased Frank-Starling mechanism. sPAP and sPO_2_ correlated fairly with RA active emptying volume which is in accordance with results of studies examining RA function of individuals with non-altitude-related pulmonary artery hypertension [23]. Of note, data of the same study cohort using the same echocardiographic methods did not indicate a significant increase in LA contractile performance [22]. In summary, these results suggest a close relation between reduced oxygen supply at high altitude and contractile RA workload demand in healthy individuals ascending to high altitudes.

There are immediate clinical implications based on our findings. Several cardiovascular conditions have profound impact on RA active emptying function and consequently compromise functional reserve. Examples include non-altitude-related pulmonary artery hypertension [23] and atrial fibrillation [24], diseases which even might be clinically silent [25]. Furthermore, aging has been associated with an increase of right atrial active emptying fraction reducing RA contractile reserve [23]. Comprehensive functional cardiac testing including exercise stress testing in normoxia as well as hypoxia prior to altitude exposure has the potential to uncover impaired functional reserve of the cardiovascular system and to identify silent pathology. Consequently, it might help to reduce high-altitude-associated complications like acute RV failure.

Furthermore, exercise, such as mountaineering and climbing, acutely increases cardiac wall stress, with the RV disproportionally affected compared to the left ventricle [26] partly due to exercise-induced pulmonary hypertension [27]. The immediate impact of exercise on RV and RA function at high altitude, which can be assessed by exercise echocardiography, remains uncertain and represents an important area of future work.

It is noteworthy that comprehensive echocardiography and cardiac stress testing are not included in the current recommendations for high-altitude exposure of individuals with pre-existing cardiovascular conditions [28]. Our results suggest that comprehensive echocardiographic assessment of the RA and RV should be taken into considerations for future revisions.

### Limitations

Invasive right-heart catheterization is considered to be the gold-standard method for the assessment of sPAP. However, it has been shown that echocardiographic and invasive measurements of sPAP are in good agreement [12]. Our participants were healthy and only few showed an exaggerated sPAP increase at high altitude. Thus, our results cannot be extended to individuals with underlying structural heart disease and/or with marked increase in sPAP. The software used in this study was originally developed for calculations of LV strain. However, the same methodology is used for the assessment of manual drawing of the region of interest in different chambers. Time point of pre-atrial contraction volume was obtained at the onset of the P-wave on surface electrocardiograms. Adding the Doppler-derived onset of the A-wave or the strain curve itself could have improved accuracy of marking this specific time point. However, since all p-waves could be identified, the added value of additional methods appears limited. For calculating RA conduit volume LV SV was used as a substitute for RV SV, as commonly practiced at low altitude. However, no comparison study exists for high altitude conditions where intracardiac shunting occurs. Since our participants were predominantly physically fit men the results cannot be directly extended to female subjects.

## Conclusions

The present study shows that active and rapid ascent of healthy individuals to 4559 m is associated with an increased contractile performance of the RA to compensate for the increased workload of the RV at high altitude. Taking into account that several cardiovascular conditions have profound impact on RA function, comprehensive functional cardiac testing prior to altitude exposure offers the potential to reduce high-altitude-associated cardiovascular problems by revealing impaired RA functional reserve and identifying silent pathology. Further studies could include comprehensive resting- and stress echocardiography under normoxic but also hypoxic conditions to improve risk stratification of subgroups who are at risk for high-altitude-induced right-heart dysfunction.

## Electronic supplementary material

Below is the link to the electronic supplementary material.

Supplementary file1 (DOCX 28 kb)
