# Supplementary material for: Preserved right ventricular function but increased right atrial contractile demand in altitude-induced pulmonary hypertension
Source: Int J Cardiovasc Imaging. 2020 Mar 9;36(6):1069–76. doi: 10.1007/s10554-020-01803-x (PMC7228961; doi:10.1007/s10554-020-01803-x)
Supplement: Supplementary file 1 — Supplementary file1 (DOCX 28 kb) [file 10554_2020_1803_MOESM1_ESM.docx]

Supplemental Table 1. *Influence of AMS on echocardiographic parameters of right atrial and ventricular function at low- and high altitude*

|  | Group | Low Alt | High Alt-7h | High Alt-20h | High Alt-44h | P*t* | P*group* | P*t*x*group* |
| --- | --- | --- | --- | --- | --- | --- | --- | --- |
| **Physiological parameters** |  |  |  |  |  |  |  |  |
| sPAP | AMS -  AMS + | 25.2 ± 5.3  23.6 ± 2.5 | 38.4 ± 10.2  37.8 ± 6.1 | 37.7 ± 11.3  38.8 ± 6.9 | 37.7 ± 8.5  38.7 ± 8.5 | <0.001 | 0.640 | 0.983 |
| **RV parameters** |  |  |  |  |  |  |  |  |
| TAPSE (mm) | AMS -  AMS + | 22.5 ± 3.5  24.7 ± 3.3 | 24.1 ± 2.9  26.1 ± 2.9 | 25.5 ± 3.0  26.0 ± 2.5 | 24.1 ± 3.0  25.8 ± 3.1 | 0.004 | 0.034 | 0.435 |
| FAC (%) | AMS -  AMS + | 50.7 ± 7.6  52.4 ± 7.4 | 48.2 ± 6.7  50.5 ± 6.9 | 50.7 ± 6.3  46.5 ± 9.7 | 48.5 ± 9.0  48.5 ± 7.9 | 0.177 | 0.981 | 0.153 |
| RV s’ (cm/sec) | AMS -  AMS + | 12.8 ± 2.1  13.0 ± 1.6 | 13.6 ± 2.1  14.6 ± 1.9 | 13.9 ± 2.3  14.0 ± 2.0 | 13.8 ± 2.1  14.6 ± 1.7 | 0.001 | 0.289 | 0.441 |
| MPI | AMS -  AMS + | 0.38 ± 0.09  0.43 ± 0.11 | 0.39 ± 0.14  0.41 ± 0.17 | 0.40 ± 0.13  0.39 ± 0.16 | 0.42 ± 0.10  0.38 ± 0.13 | 0.997 | 0.903 | 0.164 |
| RV Strain (%) | AMS -  AMS + | 24.8 ± 4.3  26.0 ± 4.6 | 24.8 ± 3.8  26.4 ± 4.4 | 26.2 ± 3.7  26.7 ± 4.0 | 26.2 ± 4.3  27.5 ± 4.6 | 0.146 | 0.361 | 0.885 |
| **RA parameters** |  |  |  |  |  |  |  |  |
| RA Reservoir Strain (%) | AMS -  AMS + | 48.9 ± 10.9  51.3 ± 9.0 | 53.7 ± 12.4  53.3 ± 9.8 | 52.8 ± 10.8  56.7 ± 13.3 | 54.3 ± 10.4  55.2 ± 13.6 | 0.075 | 0.554 | 0.724 |
| RA Conduit Strain (%) | AMS -  AMS + | 30.0 ± 10.0  31.9.6 ± 7.8 | 27.3 ± 9.9  28.6 ± 10.2 | 29.0 ± 11.1  30.5 ± 8.9 | 31.1 ± 10.8  32.5 ± 10.5 | 0.128 | 0.540 | 0.997 |
| RA Contraction Strain (%) | AMS -  AMS + | 19.0 ± 7.1  19.4 ± 5.2 | 26.4 ± 12.0  24.7 ± 7.8 | 24.3 ± 6.4  26.3 ± 10.8 | 23.2 ± 6.2  22.7 ± 9.1 | <0.001 | 0.980 | 0.676 |

Values are presented as arithmetic mean ± SD. sPAP: systolic pulmonary artery pressure; TAPSE, tricuspid annular plane systolic excursion; FAC, Fractional area change; s’, pulsed-wave Doppler tissue imaging (DTI)-derived peak systolic myocardial velocity (averaged from basal septum and basal lateral wall); MPI, myocardial performance index; RV, right ventricle; RA, right atrial; Low Alt = low altitude (424 m); High alt = high altitude (4559 m); * indicates statistical significance defined as *p* < 0.05.
